# SELF-EXTERMINATION ATTEMPTED THROUGH THE 128 NAILS INTAKE

**DOI:** 10.1590/0102-6720201600030020

**Published:** 2016

**Authors:** Juliana L. LUSVARGHI, Marcelo C. FATURETO

**Affiliations:** Universidade Federal do Triângulo Mineiro - UFTM, Uberaba, MG, Brazil

**Keywords:** Suicide, Teenager, Nail

## INTRODUCTION

Suicide is among the top ten causes of death in all age groups and with higher incidence between 15 and 35 years. Its incidence is increasing in young population^7^.

According to the World Health Organization, various stress conditions can increase the risk of suicide^1^. Eighty-five percent of patients who ingest foreign body have previous psychiatric illness and 84% of these patients have had previous intakes^5^.

From ingested foreign bodies 90% pass spontaneously through the gastrointestinal tract; 10-20% requires endoscopic removal; and 1% surgical approach^6^. In the general population, the foreign bodies are more often accidentally ingested such as bones, thorns or fruit stones. Most are housed in the physiological constrictions of the esophagus or abnormal narrowing sites (stenosis, rings or malignant tumors).

Here is presented one case of self-extermination attempt with continuous intake of nails in the course of a year.

## CASE REPORT

**FIGURE 1:**
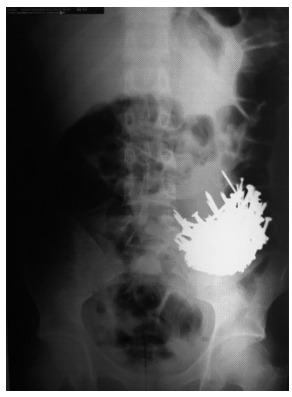
Abdominal radiograph showing strange body image in the left iliac fossa

Teenager of 16 year old was admitted with nails intake history during one year claiming attempt to self-extermination after constant arguments with his father and continuous nails intake. The parents were scavengers and had woodwork in which the patient had free access to the ingested material. Two days of admission he had epigastric pain, vomiting, and an episode of blackened stools. Physical examination showed good general condition, no collaborative, pallid (1+ / 4+), emaciated, heart beat 105 bpm, blood pressure of 120x80 mmHg, flat and flaccid abdomen, painful to deep palpation of epigastrium and no sudden pain to decompression. A large number of nails in the left iliac fossa was seen in abdominal radiograph (Figure 1); blood count was with leukocytosis and left shift.

**FIGURE 2:**
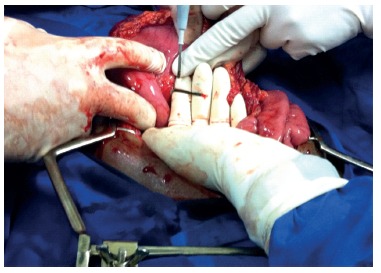
Blocked perforation on gastric rear wall with output of one nail

**FIGURE 3:**
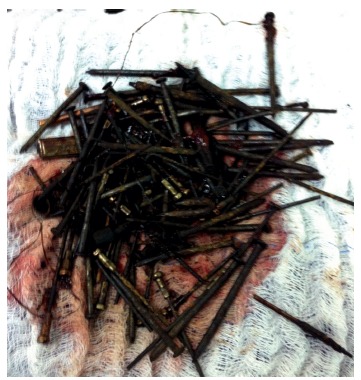
Total of 128 nails removed after gastric/jejunal opening

Laparotomy was indicated with bolus palpation of nails in the stomach and blocked perforation on the rear wall with output of one nail (Figure 2). Debridement procedure was done followed by gastrorraphy of the rear wall, and gastrotomy withdrawing 127 nails (Figure 3) with approximate size of 15 cmx15 mm. It was chosen further realization of fluoroscopy showing one nail in proximal jejunum removed by jejunotomy. 

Liquid diet initiated on the second day after surgery. He was discharged on the seventh day as outpatient. He was conducted to Guardianship Council and psychiatric evaluation before leaving the hospital and were prescribed Haldol, Phenergan and Fluoxetine; he was lost of medical assistance after that. Later contact with his mother, she was apprehensive about his attitudes and another suicide attempt; he was aggressive with the other five brothers. The Guardianship Council assessed the case and due to the conditions was chosen to put him into hospital care for two years until he gets adulthood.

## DISCUSSION

Foreign body ingestion is common in the pediatric population and the majority of victims are children and infants. Adults are located in three groups: psychological or suicide, manipulators or accidental ingestion^1^ ^,^ ^3^ ^,^ ^4^ ^,^ ^5^. Foreign bodies impacted in the esophagus can cause obstruction or perforation with consequent pneumothorax, mediastinitis or pericarditis. In the stomach, the most common complications include perforation, infection, peritonitis, unexplained fever, vomiting, abdominal pain and hematochezia. The diagnosis is mainly with abdominal radiograph if the object is radiopaque, and if radiolucent, can it be made with ingestion of small amount of barium contrast. On suspicion of perforation, it is contraindicated the use of barium. Endoscopy is the more often used exam, and although diagnostic it can also be therapeutic in most cases. Conservative treatment can be adopted in blunt objects with a diameter of <2.5 cm^5^.
